# New Delhi Metallo-β-Lactamase–producing *Enterobacteriaceae*, United States

**DOI:** 10.3201/eid1906.121515

**Published:** 2013-06

**Authors:** J. Kamile Rasheed, Brandon Kitchel, Wenming Zhu, Karen F. Anderson, Nancye C. Clark, Mary Jane Ferraro, Patrice Savard, Romney M. Humphries, Alexander J. Kallen, Brandi M. Limbago

**Affiliations:** Centers for Disease Control and Prevention, Atlanta, Georgia, USA (J.K. Rasheed, B. Kitchel, W. Zhu, K.F. Anderson, N.C. Clark, A.J. Kallen, B.M. Limbago);; Massachusetts General Hospital, Boston, Massachusetts, USA (M.J. Ferraro);; Johns Hopkins University, Baltimore, Maryland, USA (P. Savard);; Johns Hopkins Health System, Baltimore (P. Savard);; University of California Los Angeles David Geffen School of Medicine, Los Angeles, California, USA (R.M. Humphries)

**Keywords:** New Delhi metallo-β-lactamase, NDM, carbapenemase, carbapenem resistance, Enterobacteriaceae, bacteria, antimicrobial resistance, United States

## Abstract

We characterized 9 New Delhi metallo-β-lactamase–producing *Enterobacteriaceae* (5 *Klebsiella pneumoniae*, 2 *Escherichia coli*, 1 *Enterobacter cloacae*, 1 *Salmonella enterica* serovar Senftenberg) isolates identified in the United States and cultured from 8 patients in 5 states during April 2009–March 2011. Isolates were resistant to β-lactams, fluoroquinolones, and aminoglycosides, demonstrated MICs ≤1 µg/mL of colistin and polymyxin, and yielded positive metallo-β-lactamase screening results. Eight isolates had *bla*_NDM-1_, and 1 isolate had a novel allele (*bla*_NDM-6_). All 8 patients had recently been in India or Pakistan, where 6 received inpatient health care. Plasmids carrying *bla*_NDM_ frequently carried *AmpC* or extended spectrum β-lactamase genes. Two *K. pneumoniae* isolates and a *K. pneumoniae* isolate from Sweden shared incompatibility group A/C plasmids with indistinguishable restriction patterns and a common *bla*_NDM_ fragment; all 3 were multilocus sequence type 14. Restriction profiles of the remaining New Delhi metallo-β-lactamase plasmids, including 2 from the same patient, were diverse.

During the past decade, there has been an emergence of carbapenem-resistant *Enterobacteriaceae* that produce carbapenemases, enzymes that efficiently hydrolyze carbapenems, as well as most β-lactam drugs (*1*). The most common carbapenemase among *Enterobacteriaceae* in the United States is the Ambler class A *Klebsiella pneumoniae* carbapenemase (KPC), an enzyme that is found throughout the United States and globally (*2*,*3*). The emergence of another group of carbapenemases, the Ambler class B metallo-β-lactamases (MBLs), is of great concern worldwide (*4*). Until recently, MBLs were rarely identified in the United States and were found exclusively in *Pseudomonas aeruginosa* (*5*). However, recent reports of producing IMP– and VIM-type MBLs *K. pneumoniae* (*6**,**7*) have increased concerns over additional transmissible carbapenem resistance mechanisms in *Enterobacteriaceae*.

Among the most recent carbapenemases to appear in the United States is the newly described New Delhi MBL (NDM) (*8*–*12*). First reported in 2009, NDM-1 was initially identified in *K. pneumoniae* and *Escherichia coli* clinical isolates obtained from a Swedish patient who had been hospitalized in India (*13*). Drug-resistant gram-negative bacteria that produce NDM have been found in community and health care settings in India and Pakistan in a wide range of gram-negative genera containing diverse *bla*_NDM_-harboring plasmids, and have been reported in >15 countries worldwide (*4*,*14*,*15*). The widespread dissemination of NDM-producing isolates and the apparent ease of mobility of *bla*_NDM_ is a major threat to public health on a global scale.

To complement reports of individual cases (*8*,*10*,*12*), we performed extensive laboratory characterization of 9 clinical isolates of NDM-producing *Enterobacteriaceae* collected from patients in the United States during April 2009–March 2011. Strain typing and plasmid restriction analysis were performed to identify common lineages. We also describe a novel NDM-encoding allele, designated *bla*_NDM-6_.

## Bacterial Strains

Nine clinical isolates (5 *K. pneumoniae*, 2 *E. coli*, 1 *Enterobacter cloacae*, and 1 *Salmonella enterica* serovar Senftenberg), were collected from 8 patients during April 2009–March 2011 and submitted to the Centers for Disease Control and Prevention (Atlanta, GA, USA) for reference susceptibility testing during January 2010–April 2011 (Table 1). Four patients were from California and 1 each was from Illinois, Maryland, Massachusetts, and Virginia. Species identification was confirmed with the Vitek 2 automated system (bioMérieux Vitek Systems Inc., Hazelwood, MO, USA). The *S. enterica* serovar Senftenberg isolate was further classified by serotyping (*12*). A previously identified NDM-1–producing *K. pneumoniae* isolate (0S-506) from Sweden (*13*) was used as a positive control for phenotypic and molecular characterization methods, including strain typing of *K. pneumoniae* isolates. As part of a public health intervention for each of these isolates, an epidemiologist from CDC contacted local health departments and providers to identify characteristics of patients from whom NDM-producing isolates were obtained.

**Table 1:** Epidemiologic information for New Delhi metallo-β-lactamase–producing isolates, United States, April 2009–March 2011*

Patient no.	Isolate no.	Organism	Date of isolation	State	Isolation site	Patient age	Patient and travel history
1	1000654	*Enterobacter cloacae*	2009 Apr	MA	Urine	65 y	Hospitalized in India before coming to United States (*8*)
2	1000527	*Klebsiella pneumoniae*	2009 Dec	CA	Urine	73 y	Hospitalized in India before returning to United States (*8*)
3	1001728	*Escherichia coli*	2010 May	IL	Urine	41 y	Chronic medical problems; traveled to India 3–4 mo before positive culture. No known hospitalizations during travel (*8*)
4	1100192	*K. pneumoniae*	2010 Sep	CA	Resp.	13 mo	Hospitalized in Pakistan 5 months before admission in United States (*10*)
5	1100101	*E. coli*	2010 Oct	VA	Resp.	67 y	Received medical care in India but not hospitalized
6	1100770	*K. pneumoniae*	2010 Dec	CA	Urine	70 y	Hospitalized for 1 mo in India before transfer to US hospital
7	1100975	*K. pneumoniae*	2011 Jan	MD	Resp.	60 y	Hospitalized in India before transfer to US hospital (*12*)
	1101168	*Salmonella enterica* serovar Senftenberg	2011 Feb	MD	Feces		
8	1101459	*K. pneumoniae*	2011 Mar	CA	Blood	57 y	Hospitalized in India; subsequently hospitalized in United States

*Resp., respiratory sample.

## Susceptibility to Selected Antimicrobial Agents

MICs of amikacin, aztreonam, cefotaxime, cefepime, ciprofloxacin, colistin, doripenem, ertapenem, gentamicin, imipenem (IMP), meropenem, polymyxin B, tetracycline, tigecycline, and trimethoprim/sulfamethoxazole were determined by using reference broth microdilution (BMD) with panels prepared in-house according to Clinical and Laboratory Standards Institute (Wayne, PA, USA) guidelines (*16*) and stored at −70°C until use. MICs of tigecycline were interpreted according to breakpoints established by the US Food and Drug Administration (Silver Spring, MD, USA) (www.rxlist.com/tygacil-drug.htm). *E. coli* ATCC 25922, *K. pneumoniae* ATCC BAA-2146, *P. aeruginosa* ATCC 27853, *Staphylococcus aureus* ATCC 29213, and *Enterococcus faecalis* ATCC 29212 were used for quality control.

## BMD Screening for Metallo-β-Lactamase

MICs in the absence and presence of a combination of 0.2 mmol/L EDTA (Sigma-Aldrich, St. Louis, MO, USA) and 0.02 mmol/L 1,10-phenanthroline (Acros Organics, Geel, Belgium) were determined as described (*17*) by using screening wells containing IMP at concentrations ranging from 0.25 µg/mL through 1,024 µg/mL. A ratio ≥4 in the IMP MIC compared with the IMP MIC in the presence of chelators (IMP+EP) was considered a positive result for MBL production. *K. pneumoniae* ATCC BAA-2146 and *P. aeruginosa* ATCC 27853 were used as positive and negative controls, respectively.

## Modified Hodge Test

The Modified Hodge test (MHT), which is recommended by Clinical and Laboratory Standards Institute as a confirmatory test for carbapenemase production (*16*), was performed for each strain with 10-µg disks containing meropenem and ertapenem (Becton-Dickinson, Sparks, MD, USA). *K. pneumoniae* ATCC BAA-1705 and BAA-1706 were used as positive and negative controls, respectively.

## Etest for Detection of MBLs

Detection of MBLs was performed with Etest MBL strips (AB bioMérieux, St. Louis, MO, USA) containing IMP (IP) and IMP + EDTA (IPI). Strips were used according to instructions provided by the manufacturer.

## Detection of *bla*_NDM_ and *bla*_KPC_ by Real-Time PCR

A multiplexed Taqman-based real-time PCR for *bla*_NDM_ and *bla*_KPC_, as well as the universal bacterial 16S rRNA–encoding gene (*18*) as an endogenous control for DNA amplification, was performed on the 7500 Fast system (Applied Biosystems, Carlsbad, CA, USA). Cell lysates were prepared as described (*19*). Each PCR (20-μL volume) included 1× QuantiFast Probe PCR Master Mix (QIAGEN, Valencia, CA, USA), a combined primer/probe solution with final concentrations of 500 nmol/L for each primer and 250 nmol/L for each probe (Table 2), and 2 μL of template. Included in each assay were a *bla*_NDM_-positive control (*K. pneumoniae* ATCC BAA-2146), a *bla*_KPC_-positive control (*K. pneumoniae* ATCC BAA-1705), a carbapenemase-negative control (*K. pneumoniae* ATCC BAA-1706), and a no template control. Cycling conditions were a 3-min enzyme activation step at 95°C, followed by 40 cycles for 3 s at 95°C and 30 s at 60°C.

**Table 2:** Sequences of primers and probes used for identification of NDM–producing isolates, United States, April 2009–March 2011*

Assay	Primers and probes	Sequence, 5′ → 3′
Real-time PCR:NDM/KPC screen	NDM, forward primer	GAC CGC CCA GAT CCT CAA
	NDM, Reverse primer	CGC GAC CGG CAG GTT
	NDM, probe (HEX)†	TG GAT CAA GCA GGA GAT
	KPC, forward primer	GGC CGC CGT GCA ATA C
	KPC, reverse primer	GCC GCC CAA CTC CTT CA
	KPC, probe (FAM)†	TG ATA ACG CCG CCG CCA ATT TGT
	16S, forward primer	TGG AGC ATG TGG TTT AAT TCG A
	16S, reverse primer	TGC GGG ACT TAA CCC AAC A
	16S, probe (CY5)†	CA CGA GCT GAC GAC AR‡C CAT GCA
DNA sequence analysis§	NDM-1 forward	ACT CGT CGC AAA GCC CAG
	NDM-1 reverse	CTC ATG TTT GAA TTC GCC C
Internal DNA sequencing primers	NDM-2F	ACA AGA TGG GCG GTA TGG A
	NDM-2R	CGT CCA TAC CGC CCA TCT
DIG-labeled probe synthesis	NDM-F1	GAA TTG CCC AAT ATT ATG CAC C
	NDM-R1	AGC GCA GCT TGT CGG CCA TG

*NDM, New Delhi metallo-β-lactamase; KPC, *Klebsiella pneumoniae* carbapenemase; DIG, digoxigenin.  †NDM probes were labeled with HEX, KPC probes were labeled with FAM, and 16S probes were labeled with CY5 at their 5′ ends. Each contained a black hole quencher at the 3′ end. ‡R, A or G (International Union of Biochemistry codes for DNA bases). §Amplification using primers NDM-1 forward and NDM-1 reverse, both located outside the coding region of *bla*_NDM_, results in a 1,013-bp product.

Reactions with 16S cycle threshold (C_t_) values of 10–30 were considered valid, those with NDM or KPC C_t_ values of 10–30 were considered NDM positive or KPC negative, and those with NDM or KPC C_t_ values ≥40 were considered NDM positive or KPC negative (www.cdc.gov/HAI/settings/lab/kpc-ndm1-lab-protocol.html).

## DNA Sequence Analysis of *bla*_NDM_

Forward and reverse primers outside the *bla*_NDM_ coding region (Table 2) were used to amplify a 1,013-bp product. Bidirectional DNA sequencing of *bla*_NDM_ was determined from independent products with primers used for amplification, as well as *bla*_NDM_ internal primers (Table 2).

## Plasmid Isolation and Transformation

Plasmid DNA was isolated from 50-mL overnight cultures by using a Plasmid Midi Kit (QIAGEN) according to the manufacturer’s protocol. To enhance the yield of large, low-copy plasmids, DNA was eluted with elution buffer prewarmed to 65°C. *E. coli* DH10BT1 competent cells (Invitrogen, Carlsbad, CA, USA) were transformed with plasmid DNA by electroporation (Gene Pulser Xcell; Bio-Rad Laboratories, Hercules, CA, USA) according to the manufacturer’s instructions. Transformants were selected on Luria-Bertani agar containing 1 µg/mL of meropenem and were screened by real-time PCR for *bla*_NDM_. Transformant plasmid DNA was evaluated by electrophoresis, and a representative transformant containing a single NDM-encoding plasmid was chosen for further study (designated by TF suffix). *E. coli* NCTC50192, which contained 4 plasmids (≈154, 66, 38, and 7 kb) (*20*), and *E. coli* V517, which contained 8 plasmids ranging from ≈56.4 kb to 2.2 kb (*21*), were used as plasmid size standards.

## Characterization of *bla*_NDM_-bearing Plasmids

Plasmid DNA from each transformant was digested with *Xmn*I (New England Biolabs, Ipswich, MA, USA), separated by electrophoresis, transferred to a nylon membrane (Zeta-Probe; Bio-Rad Laboratories), and hybridized with an 808-bp digoxigenin (DIG)–labeled *bla*_NDM_ probe (Table 2) by using the PCR DIG Probe Synthesis Kit (Roche Applied Science, Mannheim, Germany). Hybridization at 42°C, washes, and detection by using a DIG Luminescent Detection Kit (Roche Applied Science) were performed according to the manufacturer’s instructions.

NDM-encoding plasmids were assigned to an incompatibility group by using PCR replicon typing as described (*22*). Additional β-lactamases that were co-transferred with each *bla*_NDM_-carrying plasmid were identified by using the Check-MDR CT101 Microarray Assay (Check-Points BV, Wageningen, the Netherlands), which detects genes encoding extended-spectrum β-lactamases (ESBLs) (TEM, SHV, and CTX-M), plasmid-mediated AmpCs (CMY, DHA, FOX, MOX, ACC, MIR, and ACT), as well as KPC and NDM (*23*). PCR was used to screen for *armA* and *rmtC* 16S rRNA methylase genes that confer resistance to aminoglycosides (*24*).

## Pulsed-Field Gel Electrophoresis

Pulsed-field gel electrophoresis (PFGE) was performed by using the CHEF mapper electrophoresis system (Bio-Rad Laboratories) with *Xba*I-digested *K. pneumoniae* and *E. coli* chromosomal DNA, as described for *E. coli* (www.cdc.gov/pulsenet/protocols.htm). PFGE patterns were compared by using the Dice coefficient and clustering by using the unweighted-pair group method with average linkages (Bionumerics version 5.10; Applied Maths Inc., Austin, TX, USA).

## Multilocus Sequence Typing

Multilocus sequence typing (MLST) was used to classify *K. pneumoniae* and *E. coli* isolates. This procedure was performed and results were interpreted according to protocols on the Institut Pasteur MLST database website (www.pasteur.fr/recherche/genopole/PF8/mlst) (*25*,*26*).

## Clinical and Epidemiologic Information

We identified *bla*_NDM_ by real-time PCR and DNA sequence analysis for 9 clinical isolates received at the Centers for Disease Control and Prevention during January 2010–April 2011 from 8 patients (Table 1) in 5 states. Two isolates, *K. pneumoniae* 1100975 and *S. enterica* serovar Senftenberg 1101168, were isolated from the same patient 1 month apart from a clinical specimen and a surveillance specimen, respectively (*12*). NDM-producing *Enterobacteriaceae* were isolated from a variety of specimen sources, including urine (4/9), respiratory samples (3/9), feces (1/9), and blood (1/9) (Table 1), and mostly represented colonization. All 8 patients (age range 13 months–73 years, median 62.5 years) had a recent travel history (within 4 months) that included India or Pakistan, during which 6 patients received inpatient medical care, and 1 received outpatient medical care. One patient was a citizen of India who traveled frequently between the United States and India. All medical exposures abroad resulted from medical problems that occurred in that country; none of the patients had traveled for the purpose of obtaining medical care (i.e., medical tourism).

## Antimicrobial Drug Susceptibility Patterns

All 9 NDM-producing isolates from the United States and *K. pneumoniae* 0S-506 from Sweden were resistant to all β-lactams tested (including carbapenems and aztreonam), ciprofloxacin, amikacin, and gentamicin, and demonstrated MICs ≤1 μg/mL for colistin and polymyxin B; 7/9 were susceptible (MIC ≤2 μg/mL) to tigecycline. Only 2 isolates were susceptible to tetracycline, and only the *S. enterica* serovar Senftenberg isolate was susceptible to trimethoprim/sulfamethoxazole (Table 3).

**Table 3:** Antimicrobial drug susceptibility profiles of NDM-producing isolates collected and *Escherichia coli* transformants, United States, April 2009–March 2011*

Isolate no.	Organism	MIC, µg/mL										Broth microdilution MBL screen result				Modified Hodge test result
		TGC	SXT	CTX	FEP	ATM	DOR	ETP	MER	IMP		IMP+EP†	Ratio	MBL		ETP/MER
0S-506	*Klebsiella pneumoniae*	≤0.5	>8	>64	>32	>64	>8	>4	>8	>32		1	≥64	+		–/–
1100770	*K. pneumoniae*	2	>8	>64	>32	>64	>8	>4	>8	32		0.5	64	+		+/–
1100975	*K. pneumoniae*	2	>8	>64	>32	>64	>8	>4	>8	32		1	32	+		+/+
1100192	*K. pneumoniae*	1	>8	>64	>32	>64	>8	>4	>8	8		≤0.5	≥16	+		+/–
1000527	*K. pneumoniae*	>4	>8	>64	>32	>64	>8	>4	>8	>32		≤0.5	>64	+		+/+
1101459	*K. pneumoniae*	2	>8	>64	>32	>64	>8	>4	8	16		≤0.5	≥32	+		+/+
1101168	*Salmonella enterica* serovar Senftenberg	1	0.5	>64	>32	>64	8	>4	8	4		≤0.5	≥8	+		+/+
1100101	*E. coli*	≤0.5	>8	>64	>32	>64	>8	>4	>8	16		1	16	+		+/–
1001728	*E. coli*	≤0.5	>8	>64	>32	16	>8	>4	>8	8		≤0.5	≥16	+		+/+
1000654	*Enterobacter cloacae*	>4	>8	>64	>32	>64	>8	>4	>8	>32		4	>8	+		+/+
0S-506	TF	≤0.5	≤0.25	>64	16	64	4	4	4	4		≤0.5	≥8	+		+/+
1100770	TF	≤0.5	≤0.25	>64	32	32	4	8	4	4		≤0.5	≥8	+		+/–
1100975	TF	≤0.5	≤0.25	>64	16	32	4	4	4	2		≤0.5	≥4	+		+/–
1100192	TF	≤0.5	≤0.25	>64	16	≤2	2	2	1	2		≤0.5	≥4	+		+/+
1000527	TF	≤0.5	≤0.25	>64	32	8	8	8	8	4		≤0.5	≥8	+		+/+
1101459	TF	≤0.5	≤0.25	>64	16	≤2	4	4	4	4		≤0.5	≥8	+		+/+
1101168	TF	≤0.5	≤0.25	>64	16	≤2	8	>8	8	8		≤0.5	≥16	+		+/+
1100101	TF	≤0.5	≤0.25	>64	>32	>64	>8	>8	16	8		≤0.5	≥16	+		+/+
1001728	TF	≤0.5	≤0.25	>64	32	32	8	8	8	8		≤0.5	≥16	+		–/+
1000654	TF	≤0.5	>8	>64	>32	>64	>8	>8	32	32		≤0.5	≥64	+		+/+
Recipient	*E. coli* DH-10BT1	≤0.5	≤0.25	≤0.12	≤0.5	≤2	≤0.12	≤0.12	≤0.12	≤0.5		≤0.5	≤1	–		–/–

*NDM, New Delhi metallo-β-lactamase; ID, identification; MBL, Ambler class B metallo-β-lactamase; TGC, tigecycline; SXT, trimethoprim/sulfamethoxazole; CTX, cefotaxime; FEP, cefepime; ATM, aztreonam; DOR, doripenem; ETP, ertapenem; MER, meropenem; IMP, imipenem; EP, chelator; TF, transformant. †IMP + EDTA + 1,10-phenanthroline, μg/mL.

## Detection of NDM Producers

Although each of the 9 isolates showed resistance to carbapenems, detection of carbapenemase activity by using the MHT was variable (Table 3). Six of 9 isolates had a positive MHT result for meropenem and ertapenem, and 3 were positive for ertapenem but negative for meropenem. *K. pneumoniae* 0S-506 was MHT negative for both carbapenems. The Etest MBL result was positive for *K. pneumoniae* 0S-506 and for 6/9 isolates from the United States (IP:IPI ratio ≥12). The remaining 3 isolates showed either a phantom zone or deformed ellipse, which are also indicative of an MBL according to the AB Biodisk information, although deformation of the ellipse can be difficult to recognize (*10*). The BMD MBL screen provided the most conclusive results for MBL detection: all 9 isolates and *K. pneumoniae* 0S-506 demonstrated an MIC IMP:IMP+EP ratio ≥8, which is indicative of MBL production (Table 3).

## Sequencing of the *bla*_NDM_ Gene

DNA sequencing of *bla*_NDM_ from each of the 9 isolates showed that 8 encoded NDM-1, but the coding sequence in *E. coli* 1100101 and its transformant differed from that of *bla*_NDM-1_ (GenBank accession no. FN396876) by a C→T modification at nucleotide position 698, resulting in an alanine→valine substitution at aa 233 in the inferred protein. This novel NDM variant has been designated NDM-6 (G.A. Jacoby and K. Bush, www.lahey.org/Studies) and its sequence has been deposited in GenBank under accession no. JN967644.

## Antimicrobial Drug Resistance Transferred on the *bla*_NDM_ Plasmid

Because the NDM-producing isolates from the United States and *K. pneumoniae* 0S-506 from Sweden contained multiple plasmids (range 3–6 plasmids), the *bla*_NDM_-bearing plasmid from each isolate was transferred to a plasmid-negative *E. coli* by electroporation. A transformant carrying only the *bla*_NDM_-bearing plasmid was chosen for further study.

Eight transformants were either resistant or intermediate to cefepime, cefotaxime, and all carbapenems tested, but transformant 1100192-TF was susceptible to meropenem (Table 3). Five transformants remained resistant to aztreonam, indicating that an additional resistance mechanism (e.g., AmpC or ESBL) was also carried on the NDM-encoding plasmid because MBLs do not independently hydrolyze aztreonam. Resistance to amikacin and gentamicin was co-transferred in 7 instances, but 1100192-TF remained susceptible to both drugs, and 1100101-TF was resistant only to amikacin. All transformants remained susceptible to ciprofloxacin and tetracycline, and only 1000654-TF showed resistance to trimethoprim/sulfamethoxazole.

The MHT identified carbapenemase activity in all transformant strains, although 3 were positive with only 1 of 2 carbapenems (Table 3). Only transformant 1000654-TF showed an Etest MBL IP:IPI ratio ≥8; the remainder had either a phantom zone or an ellipse deformation. The BMD MBL screening method detected MBL production in all transformants (Table 3).

Additional β-lactamase genes carried in parental strains and transformants were identified by using the Check-MDR CT101 microarray assay (*23*) (Table 4). Three transformants that remained susceptible to aztreonam had only the NDM β-lactamase. Transformants with aztreonam resistance carried either a CMY-II-type AmpC (n = 4) or a CTX-M-1-type ESBL (n = 2), in addition to *bla*_NDM_.

**Table 4:** Antimicrobial drug resistance determinants detected in clinical isolates and transformants, and incompatibility group assignment of *bla*_NDM_–bearing plasmids, United States, April 2009–March 2011*

Determinant	*Kp* 0S-506		*Kp* 1100770		*Kp* 1100975		*Kp* 1100192		*Kp* 1000527		*Kp* 1101459		*Sal* 1101168		*E. coli* 1100101		*E. coli* 1001728		*E. clo* 1000654		*E. coli* recip.
β-lactam resistance†	I	TF	I	TF	I	TF	I	TF	I	TF	I	TF	I	TF	I	TF	I	TF	I	TF	
*bla*_CTX-M-1-Type_	+	–	+	–	+	–	+	–	+	–	+	–	–	–	+	+	–	–	+	+	–
*bla*_CMY-II-Type_	+	+	+	+	+	+	+	–	+	+	–	–	+	–	+	–	+	+	–	–	–
*bla*_NDM_	+	+	+	+	+	+	+	+	+	+	+	+	+	+	+	+	+	+	+	+	–
Aminoglycoside resistance‡																					
* armA*	+	+	+	+	+	+	ND	ND	–	–	–	–	+	+	–	–	–	–	+	+	–
* rmtC*	–	–	–	–	–	–	ND	ND	+	*+*	–	–	–	–	–	–	+	+	–	–	–
Plasmid replicon‡		A/C		A/C		A/C		UT		A/C		UT		L/M		FII		A/C		FII	

*Kp, *Klebsiella pneumoniae*; *Sal*, *Salmonella enterica* serovar Senftenberg; *E. coli*, *Escherichia coli*; *E. Clo*, *Enterobacter cloacae*; recip, recipient; I, isolate; TF, *E. coli* DH-10BT1 transformant containing a single New Delhi metallo-β-lactamase–encoding plasmid; +, target detected by the assay; –, target not detected by the assay; ND, not done (aminoglycoside resistance was not transferred); UT, untypeable by PCR replicon typing (*22*). †Detected by using the Check-MDR CT101 microarray assay (*23*). ‡Detected by PCR.

Four of 8 transformants resistant to amikacin and gentamicin contained *armA*, and 2 contained *rmtC* (Table 4), both of which are 16S rRNA methylase genes that confer high-level resistance to nearly all aminoglycosides (*24*). The mechanism conferring aminoglycoside resistance in the remaining 2 transformants was not caused by *armA* or *rmtC* (Table 4) and was not characterized further.

## *bla*_NDM_-bearing Plasmids

**Figure 1:**
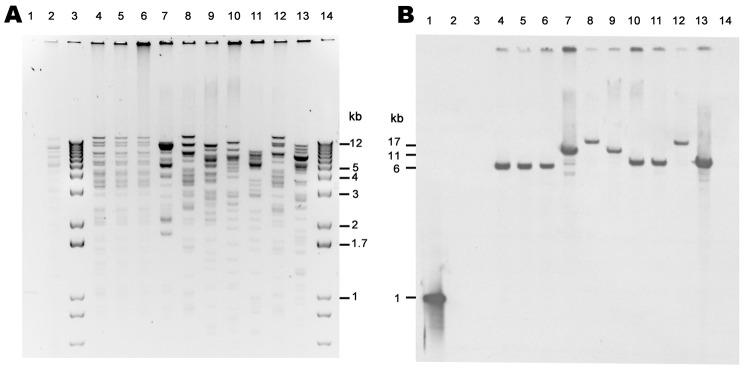
*Xmn*I restriction analysis of New Delhi metallo-β-lactamase (NDM)–encoding plasmids, United States, April 2009–March 2011, from transformants (A) and subsequent Southern blot analysis with digoxigenin-labeled *bla*_NDM_ probe hybridized to a blot of same gel (B). Lane 1, NDM PCR product, positive control; lane 2, NDM-negative plasmid (ATCC-1705); lanes 3 and 14, 1-kb plus marker; lane 4, TF 0S-506; lane 5, TF 1100770; lane 6, TF 1100975; lane 7, TF1100192; lane 8, TF 1000527; lane 9, TF 1101459; lane 10, TF 1101168; lane 11, TF 1100101; lane 12, TF 1001728; lane 13, TF 1000654.

The incompatibility groups of NDM-encoding plasmids were primarily A/C (n = 4), but also included FII (n = 2), L/M (n = 1) and 2 plasmids that were untypeable (Table 4). Eight *Xmn*I restriction patterns were observed among the NDM-encoding plasmids isolated from transformants of the isolates from the United States and isolate 0S-506 from Sweden (Figure 1). Plasmid restriction profiles from *K. pneumoniae* 0S-506 and 2 *K. pneumoniae* isolates (1100770 and 1100975) were indistinguishable. Each isolate carried *bla*_NDM_ on an *Xmn*I fragment of ≈6 kb (Figure 1) and had similar transferred antimicrobial susceptibility profiles (Table 3); carried the same ESBL and *AmpC* genes; and had plasmid replicon type A/C (Table 4). Other *bla*_NDM_-bearing plasmids were diverse, including those isolated from the same patient (*K. pneumoniae* 1100975 and *S. enterica *serovar senftenberg 1101168) (Figure 1).

## Strain Typing

**Figure 2:**
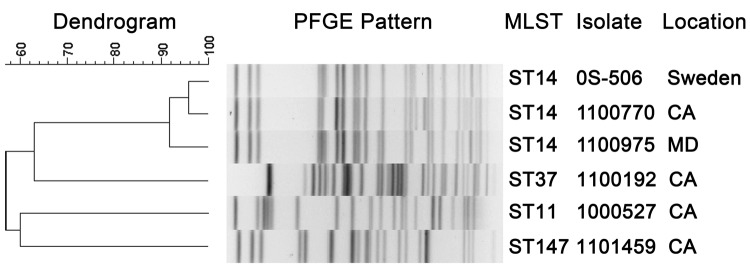
Dendrogram showing pulsed-field gel electrophoresis (PFGE) analysis and multilocus sequence typing (MLST) results for New Delhi metallo-β-lactamase–producing *Klebsiella pneumoniae* isolates, United States, April 2009–March 2011. Scale bar indicates % similarity. CA, California; MD, Maryland.

The *K. pneumoniae* isolates with indistinguishable *bla*_NDM_ plasmid profiles were closely related by PFGE, and all were classified as sequence type (ST)14 by MLST (Figure 2) (*13*). The remaining *K. pneumoniae* (Figure 2) and *E. coli* (data not shown) isolates showed more diverse PFGE patterns and MLST types, including ST37, ST11, and ST147. *E. coli* isolates were identified as ST500 and ST43. For most isolates, ST43 corresponds to ST131 in the MLST scheme of Wirth et al. (*27*) (S. Brisse, pers. comm.).

## Conclusions

The 9 NDM-producing isolates described were resistant to all β-lactams, including aztreonam, as well as all commonly used aminoglycosides and fluoroquinolones. In addition to NDM, each isolate carried >1 other β-lactamase, including CMY-II-type AmpCs and/or CTX-M-1-type ESBLs (which co-transferred with NDM for all but 3 isolates). Most *bla*_NDM_-bearing plasmids also carried *armA or rmtC* 16S rRNA methylase genes, which confer high-level resistance to most aminoglycosides and are often associated with these plasmids (*24*,*28*). Although resistance to ciprofloxacin and tetracycline did not transfer with the *bla*_NDM_-bearing plasmid, trimethoprim/sulfamethoxazole resistance was conferred to 1 transformant. For several strains, the transformant displayed decreased carbapenem resistance compared with a parental strain (e.g., 1100192-TF), suggesting that additional mechanisms (e.g., AmpC and ESBL) present in the parental strain and not carried on the NDM-encoding plasmid may have contributed to the initial carbapenem-resistant *Enterobacteriaceae* phenotype observed. These findings emphasize the diversity of resistance mechanisms carried on NDM-encoding plasmids, as reported (*28*).

We used 3 screening methods for phenotypic detection of MBL activity: MHT, Etest MBL, and BMD MBL. The MHT was not sensitive for detection of NDM activity; 3 isolates were positive only for 1 carbapenem tested, and *K. pneumoniae* 0S-506, the first characterized NDM-producing isolate (*13*), was negative for both carbapenems. Etest MBL definitively identified 6 parental isolates and 1 NDM-producing transformant as MBL producers, but 3 parental strains and 8 transformants displayed a phantom zone or slight deformation of the IP or IPI ellipse. The Etest MBL package insert states that these findings are indicative of an MBL, but the ellipse deformation we observed was subtle and less dramatic than the example provided. The BMD MBL screen provided the most unambiguous results, and yielded IMP to IMP + EP MIC ratios ≥8 for all NDM-producing parental and transformant strains. In an earlier validation study, this BMD MBL screen had a sensitivity of 95% and a specificity of 100% (*29*).

We reliably detected *bla*_NDM_ with a novel multiplexed real-time PCR designed to detect the *bla*_NDM_ and *bla*_KPC_ genes. DNA sequence analysis confirmed the PCR results and identified the *bla*_NDM_ allele in each isolate. One isolate contained a variant allele designated *bla*_NDM-6_. An NDM-6–producing *E. coli* strain was also recently identified in a patient in New Zealand who had received medical care in India (*30*).

Plasmids carrying *bla*_NDM_ have been reported to range in size from 50 through 400 kb (*14*,*15*). Because all isolates in this report carried multiple plasmids, it was necessary to transfer the NDM-encoding plasmid to a plasmid-negative recipient for analysis. Three *K. pneumoniae* isolates, including the original NDM-producer from Sweden (*13*), contained an ≈170-kb *bla*_NDM_–bearing plasmid, and each isolate was indistinguishable by restriction analysis and Southern blot. These strains were also closely related by PFGE and MLST (ST14). Furthermore, the antimicrobial drug susceptibility profiles of their parental and transformant isolates were similar. In contrast, the remaining isolates contained different *bla*_NDM_-bearing plasmids ranging in size from 100 kb through 200 kb, carried *bla*_NDM_ on different restriction fragments, and were not related by PFGE or MLST. Most of the *bla*_NDM_-bearing plasmids belonged to incompatibility groups A/C or L/M, both broad host range plasmids, and FII, a narrow host range plasmid (*31*). All 3 replicon types have been found to be associated with a variety of β-lactam resistance mechanisms in *Enterobacteriaceae* (*32*). These findings were consistent with reports of extensive diversity among *bla*_NDM_-bearing plasmids in *Enterobacteriaceae* (*14*).

The MLST types identified among NDM-producing *K. pneumoniae* described here have been associated with various resistant strains of *K. pneumoniae* worldwide (*28*,*33*). ST11, ST147, and ST15, a single locus variant of ST14, have been identified as epidemic clones of CTX-M-15–producing *K. pneumoniae* in Hungary (*34*). In addition, ST11 is the dominant KPC-producing strain in China (*35*) and is a single locus variant of ST258, the dominant KPC-producing strain in the United States (*2*). ST11 NDM-producing *K. pneumoniae* strains were among the first NDM-producing *Enterobacteriaceae* reported in New Zealand (*30*), and ST147 NDM-producing *K. pneumoniae* isolates have been reported in Switzerland (*28*), Canada (*36*), Australia (*37*), and in an Iraqi patient transferred to a hospital in France (*28*). ST14 has been identified among KPC-producing strains in the United States (*38*), and is associated with NDM-producing *K. pneumoniae* isolates in Kenya and the Sultanate of Oman (*28*), and as the most frequently encountered ST in a recent study of NDM-1–producing *K. pneumoniae* from 3 countries (*33*). We also report a ST43/ST131 NDM-producing *E. coli* strain in our study. This clone is most notably associated with the global dissemination of the CTX-M-15 ESBL (*39*).

Each patient associated with the isolates described here had recently been in India or Pakistan, and most had received inpatient medical care in those countries. The link between NDM acquisition and health care exposure abroad has been extensively described (*4*,*15*,*40*). However, 1 patient only had outpatient health care during travel, and another had no documented health care, although the second patient had several active medical problems, including the presence of an invasive medical device during travel. In contrast to the early NDM case-patients reported in the United Kingdom (*15*), none of the patients in our study had traveled specifically for the purpose of obtaining medical care.

Several factors contribute to the global dissemination of *bla*_NDM_ as it spreads through a variety of plasmids and bacterial strains. The environmental and epidemiologic factors driving this spread and the molecular mechanisms by which it disseminates are not well understood. However, in the 3 years since its initial description, NDM has spread rapidly worldwide and has now been described in >15 countries in 5 continents (*4*,*8*). Since the completion of this study, numerous additional NDM-producing *Enterobacteriaceae* have been identified in the United States. The relative ease with which this resistance mechanism appears to move within and between different bacterial genera, as well as mobility of humans infected or colonized with NDM producers, serves to highlight the need for reliable and rapid means of detecting drug-resistant organisms to implement infection control measures to prevent further dissemination.
